# Tumor microenvironment and immunotherapy for triple-negative breast cancer

**DOI:** 10.1186/s40364-024-00714-6

**Published:** 2024-12-31

**Authors:** Zijie Guo, Ziyu Zhu, Xixi Lin, Shenkangle Wang, Yihong Wen, Linbo Wang, Lili Zhi, Jichun Zhou

**Affiliations:** 1 Department of Surgical Oncology, Affiliated Sir Run Shaw Hospital, Zhejiang University School of Medicine, No.3 East Qingchun Road, Hangzhou, 310016 Zhejiang China; 2 Biomedical Research Center, Key Laboratory of Biotherapy of Zhejiang Province, Hangzhou, 310016 Zhejiang China

**Keywords:** Triple-negative breast cancer (TNBC), Immunotherapy, Tumor microenvironment (TME), Immune checkpoint, Cancer treatment protocols, Biomarkers

## Abstract

Triple-negative breast cancer (TNBC) is a subtype of breast cancer known for its high aggressiveness and poor prognosis. Conventional treatment of TNBC is challenging due to its heterogeneity and lack of clear targets. Recent advancements in immunotherapy have shown promise in treating TNBC, with immune checkpoint therapy playing a significant role in comprehensive treatment plans. The tumor microenvironment (TME), comprising immune cells, stromal cells, and various cytokines, plays a crucial role in TNBC progression and response to immunotherapy. The high presence of tumor-infiltrating lymphocytes and immune checkpoint proteins in TNBC indicates the potential of immunotherapeutic strategies. However, the complexity of the TME, while offering therapeutic targets, requires further exploration of its multiple roles in immunotherapy. In this review, we discuss the interaction mechanism between TME and TNBC immunotherapy based on the characteristics and composition of TME, and elaborate on and analyze the effect of TME on immunotherapy, the potential of TME as an immune target, and the ability of TME as a biomarker. Understanding these dynamics will offer new insights for enhancing therapeutic approaches and investigating stratification and prognostic markers for TNBC patients.

**Supplementary Information:**

The online version contains supplementary material available at 10.1186/s40364-024-00714-6.

## Introduction

Breast cancer (BC) is one of the most prevalent form of cancer worldwide, prompting extensive research due to its rising morbidity and mortality rates [1]. Triple-negative breast cancer (TNBC) is a distinct subtype characterized by the absence of estrogen receptor (ER), progesterone receptor (PR), and human epidermal growth factor receptor 2 (HER2) expression [2]. This subtype exhibits aggressive basal-like features, significant heterogeneity, and a poor prognosis [3]. The heterogeneity of TNBC is evident in its gene expression profiles and clinical presentations. TNBC can be further categorized into subtypes based on gene expression profiles, including Basal-like, Immunomodulatory, Mesenchymal, and Luminal Androgen Receptor (LAR) subtypes [4]. Notably, the Immunomodulatory subtype shows significant immune gene expression characteristics, suggesting a potentially enhanced response to immunotherapy [5]. TNBC lacks specific targets or biomarkers, making it called a diverse group of diseases with limited biological understanding [6]. TNBC is resistant to traditional endocrine and HER2-targeted therapies, resulting in a poor prognosis. Progress in targeted therapy for TNBC has lagged advancements in other breast cancer subtypes. At present, treatment for TNBC primarily relies on surgery, chemotherapy, and radiotherapy. However, the emergence of immunotherapy, particularly the successful use of immune checkpoint inhibitors in various cancers, has prompted research into its potential for TNBC treatment [7].

Cancer immunotherapy aims to enhance the body’s immune system to attack and kill tumor cells and improve the tumor microenvironment (TME) [8]. Unlike conventional therapies, immunotherapy is based on a comprehensive understanding of the tumor immune escape mechanism, where tumor cells evade immune surveillance and attack through various pathways to survive and proliferate [9]. This therapeutic approach has demonstrated potential long-term efficacy and tolerability in a wide range of cancers, becoming a crucial method of cancer treatment [10]. However, monotherapy of immunotherapy has limited efficacy and is prone to immune escape and drug resistance due to factors such as the TME [11]. Therefore, combination therapies that integrate targeted agents with immunotherapy and chemotherapy are emerging as a promising strategy to overcome these limitations and enhance treatment outcomes. In breast cancer, combination therapies incorporating immune checkpoint inhibitors (ICIs) alongside chemotherapy and radiotherapy have yielded favorable therapeutic effects in TNBC patients [11]. Studies have shown that combining targeted therapies, such as COX1/2 inhibitors and PI3K-γ inhibitors, with immune checkpoint inhibitors and chemotherapy can enhance the anti-tumor immune response and improve outcomes in TNBC by reprogramming the TME [12, 13].

The TME refers to the complex ecosystem of tumor cells along with their surrounding stromal cells, immune cells, blood vessels, and extracellular matrix, etc. The main components in the TME include cancer-associated fibroblasts (CAFs), tumor-associated macrophages (TAMs), lymphocytes, myeloid-derived suppressor cells (MDSCs), and various cytokines and chemokines [14]. The TME plays a key role in tumorigenesis, development, and metastasis, influencing cancer progression by regulating processes such as tumor cell growth, immune escape, and angiogenesis [15, 16]. The significance of the TME in tumor progression and treatment has been demonstrated in various cancers [17]. In breast cancer, the TME not only promotes tumor invasion and metastasis in several ways but also affects patient response to treatment and prognosis. By intervening in the TME, immunotherapy can modify the interaction between breast cancer and its microenvironment, thereby enhancing anti-tumor immune responses [18]. Moreover, in breast cancer, immune cell components of the TME, such as tumor-infiltrating lymphocytes (TILs), are considered important predictors of patient prognosis. This provides a crucial foundation for further studies on TNBC immunotherapy, indicating the potential to enhance therapeutic efficacy by modulating the TME [19–21].

Therefore, studying the TNBC tumor microenvironment is important for the prognosis and immunotherapy of TNBC. TNBC urgently needs new therapeutic strategies due to its unique biological characteristics and limited therapeutic options. As the understanding of the biology of TNBC grows, researchers have shifted their focus more towards “positive” biomarkers in TNBC [7]. Thus, immunotherapy, especially TME-related interventions, shows promise for significantly improving the prognosis of TNBC patients. It is believed that this approach will be the future of TNBC treatment. In this article, we will discuss in detail the latest advances in immunotherapy for TNBC and the specifics of TME for TNBC, and on this basis, we will delve deeper into the pathways and mechanisms of TME itself as well as its interactions with tumor cells during the development and treatment of TNBC, with the aim of further promoting the immunotherapeutic benefits of TNBC.

## Immunotherapy for TNBC

### Mechanisms of immunotherapy

In recent years, with the in-depth study of tumor immune escape mechanisms, immunotherapy has achieved significant progress, leading to the development of various immunotherapeutic modalities, including immune checkpoint inhibitors (ICIs), cancer vaccines, adoptive cell therapy (e.g., CAR-T cell therapy), and cytokine therapy [22].

**Fig. 1:**
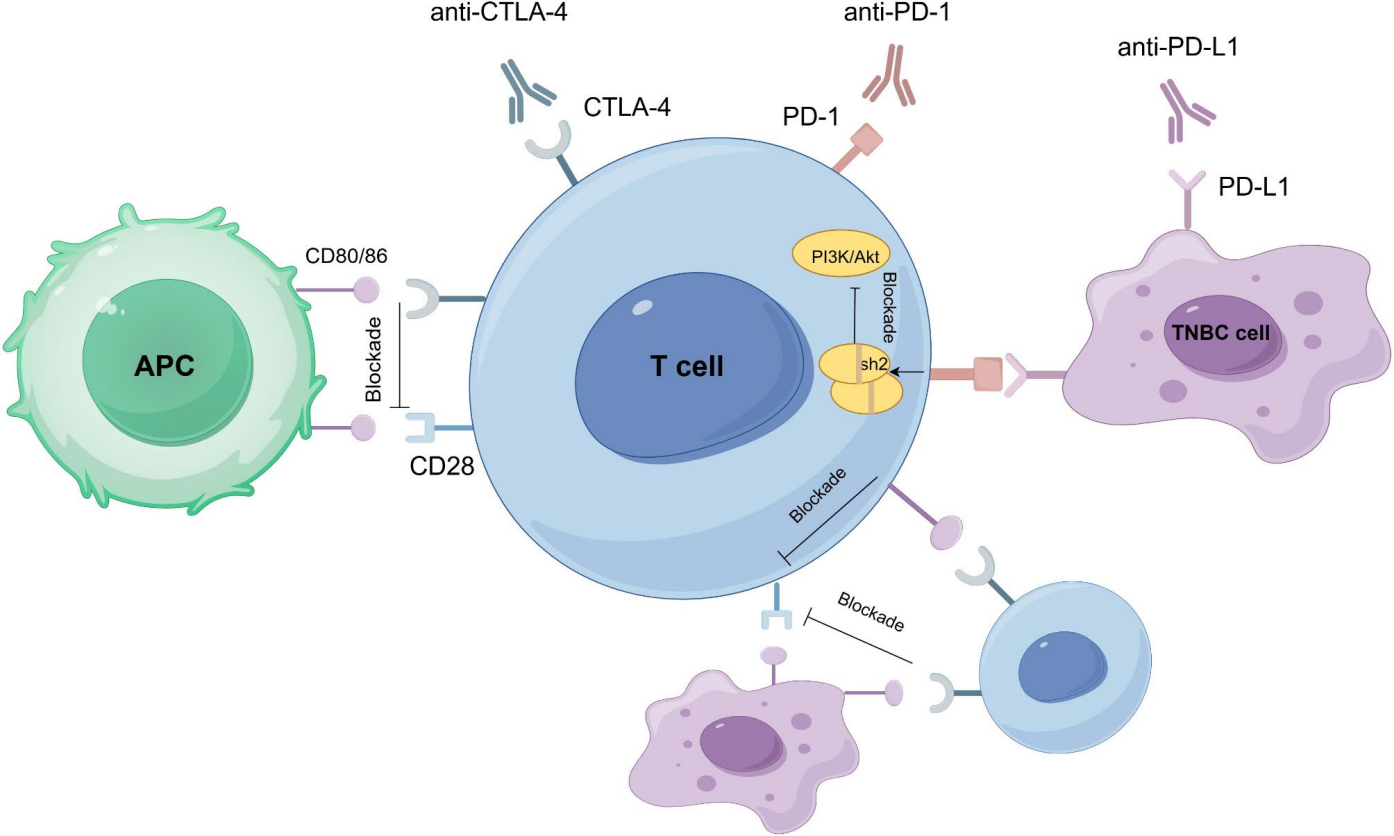
Mechanisms of ICIs

ICIs are the cornerstone of current immunotherapy, primarily targeting PD-1, PD-L1, and CTLA-4. Immune checkpoints regulate the immune system, maintaining immune homeostasis and preventing excessive immune responses by inhibiting T cell activation and function. However, tumor cells can exploit these checkpoints to evade the immune system. Immune checkpoint inhibitors have shown significant efficacy in various cancers by blocking these inhibitory signals and restoring the anti-tumor activity of T cells [23, 24]. PD-1 (Programmed cell death protein 1) is an immune-suppressing receptor on immune cells, includes T cells, NK cells, etc. When it binds to PD-L1, which is highly expressed on the surface of tumor cells, it triggers the expression of SH2-containing protein tyrosine phosphatase 2 (SHP2), which in turn inhibits the PI3K/AKT axis. This leads to inhibition of T-cell function and induction of T-cell apoptosis, preventing effective killing of tumor cells [23, 25]. PD-1 and PD-L1 inhibitors restore T cell anti-tumor function by blocking this pathway [26, 27]. CTLA-4 (Cytotoxic T-lymphocyte-associated protein 4) is another crucial immune checkpoint that acts as a suppressor of the adaptive immune response. It interacts with B7 molecules (including CD80/CD86) on kinds of cells such as T cells and antigen-presenting cells (APCs), competitively inhibiting the binding of CD28 on T cells to CD80/CD86, thereby suppressing T cell activation. These mechanisms enable CTLA-4 to hinder T-cell growth, differentiation, and the production of cytokines like IL-2, ultimately reducing anticancer immunity in the tumor microenvironment [28–30]. CTLA-4 inhibitors boost T-cell activation and antitumor responses by disrupting this pathway [31] (Fig. 1).

This figure shows the mechanism by which ICIs play a role in the process of tumor immunity. Currently used ICIs mainly target three immune checkpoints, PD-1, PD-L1 and CTLA-4. Tumor cells inhibit the function of T cells by activating the PD-1/PD-L1 and CTLA-4 signaling pathways to avoid being recognized and cleared by the immune system. In the PD-1/PD-L1 pathway, PD-1 binds to PD-L1 on the surface of tumor cells and triggers the expression of SHP2, which inhibits PI3K/AKT signaling, leading to decreased T cell function and apoptosis. And CTLA-4 blocks T cell activation by competitively inhibiting T cell binding to B7 molecules on APC. Immune checkpoint inhibitors restore the anti-tumor function of T cells by blocking these inhibitory signals. APC, antigen-presenting cells; CTLA-4, cytotoxic T-lymphocyte-associated protein 4; PD-1, programmed cell death protein 1; PD-L1, programmed death-ligand 1; TNBC, triple-negative breast cancer.

Adoptive cell therapy is a treatment that amplifies enough amounts of immune cells with anti-tumor activity by engineering immune cells in the TME and enhancing cellular immune function in patients to improve anti-tumor effects [32]. In the TME, tumor killer cells such as T cells and NK cells are often inhibited or depleted due to the presence of immunosuppressive mechanisms and environmental factors like hyperoxia and hypoxia. Adoptive cell therapy can alter the number and status of immune cells in the TME. It can not only directly eliminate the tumor but also activate the body’s immune function to help inhibit the tumor growth, leading to a relative dynamic balance within the unique tumor microenvironment, preventing further progression [33]. Cancer vaccines are designed based on tumor-specific antigens and can boost anti-tumor effects by transferring other biological elements, such as antigens in cancer cells or TMEs, to the host, triggering specific immune responses in patients. Typically, tumor whole cell vaccines contain a wide range of tumor-associated antigens, epitopes rich in CD8 T cells, CD4 helper T cells, and can present MHC class I and II restricted antigens simultaneously, promoting a comprehensive and effective anti-tumor response and generating long-lasting memory T cells. As technology advances, antibody tumor vaccines, tumor peptide vaccines, and genetic engineering vaccines are also gradually gaining approval for clinical cancer treatment [34, 35].

### Current status of immunotherapy for TNBC

Due to the absence of targets such as ER, PR, and HER2 in TNBC, conventional targeted therapies are less effective. Consequently, immunotherapy, especially ICIs, has gained significant attention for treating TNBC. PD-1 and PD-L1 inhibitors are the ICIs that have received the most focus. Preclinical studies support their role in TNBC, demonstrating that PD-1 and PD-L1 inhibitors can effectively activate immune cells and enhance their ability to specifically kill TNBC cells, significantly inhibiting tumor growth [36]. PD-1 inhibitors like pembrolizumab and nivolumab, as well as PD-L1 inhibitors like atezolizumab and durvalumab, have shown significant efficacy in clinical trials and have been approved by the FDA for use in combination therapies for TNBC [37]. The phase I KEYNOTE-012 trial demonstrated that pembrolizumab improved the objective response rate (ORR) in TNBC patients and had a favorable safety profile [38].

Beyond PD-1/PD-L1 inhibitors, other immunotherapies for TNBC are being explored and validated. CTLA-4 inhibitors, another class of ICIs, have the potential to activate or restore T-cell function in TNBC. Pabolizumab is believed to be effective in the adjuvant treatment of early TNBC. A preliminary clinical study also concluded that combining preoperative tumor cryoablation with ipilimumab shows potential intra-tumor and systemic immune effects [39]. Tumor vaccines are also being investigated for TNBC therapy, with several studies suggesting their potential value [39, 40]. Cellular immunotherapy and bispecific antibody therapy are also novel immunotherapies currently under investigation. These therapies have shown positive impacts in TNBC models in both in vitro and animal experiments, indicating a promising future for their clinical application [41–43].

However, monotherapy of immunotherapy such as PD-1/PD-L1 inhibitors in TNBC may have limited efficacy and is prone to resistance due to factors such as the TME [11]. Thus, current clinical studies focus on the efficacy of immunotherapy in combination therapy. Several studies have shown that combining ICIs with conventional chemotherapy significantly improves the prognosis for TNBC patients (Table 1). The phase III KEYNOTE-355 study revealed that pembrolizumab combined with chemotherapy significantly improved progression-free survival (PFS) and overall survival (OS) in patients with refractory TNBC [44]. The IMpassion130 study showed that atezolizumab combined with chemotherapy significantly improved PFS and OS in patients with PD-L1-positive metastatic TNBC [45]. Additionally, several clinical studies validate the efficacy of PD-1 and PD-L1 inhibitors in the neoadjuvant treatment of TNBC. The phase III KEYNOTE-522 study evaluated the efficacy of pembrolizumab combined with neoadjuvant chemotherapy in early-stage TNBC and showed that this combination improved the pathological complete response (pCR) rate and significantly prolonged event-free survival (EFS) [46]. Furthermore, the combination of PD-1/PD-L1 inhibitors with other therapeutic modalities has shown effectiveness in TNBC. One phase II clinical study found that combining radiotherapy with pembrolizumab improved the ORR in patients with metastatic TNBC [47]. Another phase II clinical study indicated that gene therapy combined with pembrolizumab significantly prolonged OS [48]. These studies, though mostly single-arm or phase II studies, also illustrate the promising future of PD-1/PD-L1 inhibitors in TNBC treatment.

**Table 1:** Clinical trials of immunotherapy for TNBC

Immunotherapeutic agents		Trials		TNBC Status		Main outcomes		Results	References
**PD-1 inhibitor**									
Pembrolizumab		KEYNOTE-355, Phase III		Advanced		PFS OS		HR for OS, 0.73; 95% CI, 0.55 to 0.95; *P* = 0.02	[49]
		NeoPACT, Phase II		Stage I to III		pCR		pCR: 58% (95% CI, 48-67%)	[50]
		TOPACIO, Phase II		Advanced or metastatic		ORR		ORR of all: 21% (90% CI, 12-33%) ORR of BRCA carriers: 7% (90% CI, 24-70%)	[51]
		KEYNOTE-086 Cohort A, Phase II		Metastatic		ORR		ORR of all: 5.3% (95% CI, 2.7-9.9%) ORR of PD-L1-positive populations: 5.7% (95% CI, 2.4-12.2%)	[52]
		KEYNOTE-086 Cohort B, Phase II		Metastatic				ORR of all: 21.4% (95% CI, 13.9-31.4%)	[53]
		KEYNOTE-012, Phase Ib		Advanced		ORR		ORR: 18.5% mPFS: 1.9 months mOS: 11.2 months	[38]
		ENHANCE 1, Phase 1b/II		Metastatic		ORR		ORR for stratum 1: 25.8% (95% CI, 15.8-38.0%) ORR for stratum 2: 21.8% (95% CI, 14.2-31.1%)	[54]
		I-SPY2, Phase II		Early		pCR		pCR: 60% vs. 22%	[55]
Nivolumab		TONIC, Phase II		Metastatic		PFS		Median PFS: 1.9 months ORR: 20%	[56]
**PD-L1 inhibitor**									
Atezolizumab		NCI-10,013, Phase II		Stages II-III		pCR		pCR: 55.6% (95% CI 40.0-70.4%)	[57]
		Impassion130, Phase III		Locally advanced or metastatic		PFS OS		HR for OS of all: 0.87 (95% CI, 0.75–1.02) *P* = 0.01 HR for OS of PD-L1 IC-positive population: 0.67 (95% CI, 0.53–0.86) *P* = 0.01	[45]
		MARIO-3, Phase II		Metastatic		Complete response		ORR: 55.3%	[58]
		Combined results from CO40151 (phase Ib) IPATunity170 (single-arm signal-seeking cohort) IPATunity130 (phase III)		Locally advanced or metastatic		ORR		ORR: 44 − 63% PFS: 5.4–7.4 months OS: 15.7–28.3 months	[59]
		IMpassion031, Phase III		Early		pCR		pCR of all: 53.4%vs. 44.2%, *P* = 0.004 pCR of PD-L1-positive population: 69%vs. 49%, *P* = 0.018	[60]
Durvalamab		BEGONIA, Phase II		Metastatic				ORR: 73%	[61]
		GeparNuevo, Phase II		Early		pCR		No significant change in pCR HR for OS: 0.48 (95% CI, 0.24–0.97) *P* = 0.036	[62]
		MEDIOLA, Phase I/II		BRCA-mutated metastatic				ORR: 58.8% mPFS: 4.9 months mOS: 20.5 months	[63]
Avelumab		JAVELIN PARP Medley, Phase 1b/II		Advanced		ORR		ORR: 18.2%(95%CI, 5.2-40.3%) Median DOR: 11.1 [3.4–20.4] months	[64]
		JAVELIN Solid Tumor, Phase 1		Locally advanced or metastatic		ORR		ORR: 5.2% ORR of PD-L1 + patients: 44.0%	[65]
**Other treatments**									
Cytokine-induced killer cell		NCT01395056		Post-mastectomy		PFS OS		Higher PFS and OS (*P* = 0.038, *P* = 0.005)	[66]
Cancer vaccine		OBI-822/821, Phase II		Metastatic				HR for PFS: 0.59 (95% CI, 0.32–1.10) *P* = 0.01	[40]

ORR, objective response rate; OS, overall survival; pCR, pathological complete response; PFS, progression free survival

Despite the significant therapeutic potential of immunotherapy in TNBC, challenges and limitations remain. Immunotherapy can trigger immune-related adverse effects (irAEs) such as skin inflammation, liver function abnormalities, and endocrine disorders. Timely monitoring and management of these effects are essential to minimize treatment-related risks [67]. Additionally, not all TNBC patients respond effectively to immune checkpoint inhibitors. Clinical data show that immunotherapy efficacy strongly correlates with PD-L1 expression levels, and response rates in PD-L1-negative patients are limited [36]. This underscores the need for identifying biomarkers for TNBC immunotherapy [68]. Moreover, the complexity of the TME, with its multiple immunosuppressive cells (e.g., tumor-associated macrophages and regulatory T cells) and inhibitory cytokines (e.g., TGF-β), poses a significant challenge for immunotherapy [69]. Therefore, an in-depth understanding of the TME and the development of effective combination therapies are crucial to enhancing the efficacy of immunotherapy in TNBC as well as the predictive value of the therapeutic prognosis.

## The tumor microenvironment and TNBC

### Characterization of the tumor microenvironment in TNBC

#### Immune cell components

The TME of TNBC has unique characteristics where cellular and environmental factors are crucial in the onset and progression of TNBC. A key feature of the TME of TNBC is the density and type of tumor-infiltrating lymphocytes (TILs) (Table 2). TNBC typically exhibits a high density of TILs, predominantly composed of CD3 + T cells and CD20 + B cells. Infiltration of CD3 + T cells is most common in TME and plays a major role. CD3 + T cells are mainly composed of CD8 + T cells and CD4 + T cells, with regulatory T lymphocytes (Tregs) and natural killer (NK) cells constituting less than 1%. The proportion of CD8 + T cells in TNBC is higher than in other breast cancer subtypes [70–72]. TILs are closely associated with the tumor’s immune escape mechanisms. Upon activation by the major histocompatibility complex (MHC) expressed on tumor cells, CD8 + T cells differentiate into cytotoxic T cells, which release IFN-γ to kill tumor cells and inhibit growth, playing a critical role in adaptive immune defense [73]. CD4 + T cells can differentiate into helper T cells and regulatory Tregs. Activated by MHC molecules, CD4 + helper T cells differentiate into subtypes such as Th1 and Th2, assisting CD8 + T cell-mediated cytotoxicity and playing an active role in tumor immunity [74]. Conversely, Tregs promote immune escape by suppressing effector T cell functions [75]. In total, a high density of TILs is often associated with a better pathological complete response (pCR) and overall survival (OS) in TNBC [20, 76]. Besides T cells, B cells occupy about 10% of the TME of TNBC, and their role and distribution have not yet been fully explored [71]. However, it is now clear that B cells tend to aggregate to form clusters of cells in the TME of breast cancer, thereby forming structures that resemble tertiary lymphoid structures [77]. B cells play an immune role mainly through antigen presentation to T cells, but their specific immune role in TNBC remains to be explored [78].

Additionally, NK cells also play a significant anti-tumor role by directly killing tumor cells and secreting cytokines like IFN-γ to inhibit tumor spread and metastasis. However, TNBC cells can evade immune surveillance by down-regulating NK cell receptors or secreting inhibitory factors [79]. Immature NK cell populations in TNBC may reduce granule-mediated cytolysis and promote metastasis and progression [80]. Despite these challenges, NK cells derived from cancer patients exhibit similar characteristics and expansion potential to those from healthy donors, with no significant difference in their oncogenic effect on TNBC cell lines [81].

Moreover, the TME of TNBC is rich in tumor-associated macrophages (TAMs), which constitute more than one-fourth of the immune cells in the TME. TAMs typically promote tumorigenesis and progression through mechanisms such as promoting cell division, inducing invasion, facilitating immune escape, and encouraging angiogenesis [82]. Previously, TAMs were classified into M1 and M2 types, with M1 TAMs enhancing anti-tumor immunity by secreting anti-inflammatory factors (e.g., IL-12 and TNF-α), and M2 TAMs inhibiting the immune response by secreting pro-inflammatory factors (e.g., IL-10 and TGF-β) and angiogenic factors like VEGF, promoting tumor growth and metastasis [83, 84]. Recent analyses have shown that TAMs in breast cancer do not strictly adhere to M1 or M2 categories but exhibit genetic characteristics of both, indicating that individual TAMs can possess both immune-promoting and suppressing functions [85, 86]. In TNBC, TAMs tend to exhibit more immunosuppressive effects, and a high abundance of TAMs is linked to poorer prognosis [87, 88]. This is likely due to the impact of TNBC’s immune profile on TAM gene expression, with a high number of macrophages in the TNBC TME enriched with genes essential for activation and antigen presentation [89].

Dendritic cells (DCs) are a minor component of the TNBC TME, but specific subpopulations, particularly migratory DCs, can exert significant antitumor effects [90]. Migratory DCs, characterized by high levels of enzymes like indoleamine 2,3-dioxygenase 1 and CD274 (PD-L1), can attract PD1-overexpressing T cells. DCs can promote anti-tumor immunity in other immune cells in the TME [91, 92]. Myeloid-derived suppressor cells (MDSCs) also play a role in the TME of TNBC. These cells weaken the host anti-tumor immune response by inhibiting the function of T cells and NK cells [93].

#### Non-immune cell components

In addition to immune cell characteristics, other environmental factors in the TME significantly influence the development of TNBC. Matrix formation, angiogenesis, and cellular metabolic processes are all crucial for cancer progression. Cancer-associated fibroblasts (CAFs) and cancer-associated adipocytes (CAAs) are highly expressed in TNBC (Table 2). CAFs promote matrix remodeling and angiogenesis, thereby supporting cancer cell proliferation, migration, and invasion [94]. In breast cancer, CAFs are divided into four subtypes - CAF-S1 to S4, with CAF-S1 and CAF-S4 enriched in TNBC, likely due to high cytokine secretion, such as IL-10 and IL-18, enhancing their ability to inhibit effector T cells [95]. CAF-S1 plays a critical role in promoting immunosuppression by altering the expression levels of immune checkpoints on CD8 + T cells and Tregs through signaling pathways like NFAT and STAT, leading to an immunosuppressive microenvironment. CAAs have multiple roles in the TME, primarily functioning as energy storage cells, providing tumor cells with ketones, fatty acids, and other metabolites to support growth. Additionally, CAAs induce cytokine secretion, such as IL and TGF, promoting breast cancer cell growth and invasion [96, 97]. CAAs can also express PD-L1, inhibiting the anti-tumor function of CD8 + T cells [98].

Angiogenesis is a crucial feature of the TME, closely related to TME hypoxia, immune response changes, and drug delivery. The most important pro-angiogenic factor in the TME of TNBC is vascular endothelial growth factor (VEGF), whose expression is highly deregulated. VEGF levels are significantly higher in the TNBC microenvironment than in other breast cancer types [99, 100]. The specialized new-born vasculature impacts the immune response in the TME, improves the hypoxic environment, and promotes tumor cell growth and migration [101]. In breast cancer, the vascular endothelium specifically expresses FAS ligand, which eliminates effector CD8 + T cells but retains Tregs [102].

**Table 2:** Main types of cells in the TME of TNBC

Types		Characteristics	Functions	Impact for patients
T cells	CD8 + T cells	Cytotoxic potential IFN-γ secretion Higher proportion in TNBC among BC subtypes	Tumor cells eradicating and anti-tumor immunity	Correlated with longer survival time in TNBC patients Potential therapeutic target
	CD4 + helper cells	/	Adjuvant CD8 + T lymphocytes, promoting tumor immunity	Potential therapeutic target
	Tregs	FOXP3 + CD25	Immune suppression of effector T cells, leading to tumor immune escape	Associated with poorer survival
B cells		Aggregation to clusters of cells Antigen presentation	Unclear in TNBC	Associated with better survival
NK cells		Cytotoxic potential Oncogenic cytokine secretion Downregulation in TNBC leading to immune escape	Tumor cells eradicating and tumor growth suppression	Potential therapeutic target
TAMs	M1-like TAMs	Secreting anti-inflammatory factors	Anti-tumor immunity	High abundance of TAMs is linked to poorer prognosis
	M2-like TAMs	Secreting pro-inflammatory factors Secreting angiogenic factors Expression of PDL1/PDL2 and CCL18	Tumor growth and metastasis promotion	
DC cells		Very low percentage of expression in TNBC PD-L1/L2 expression capacity	Promoting tumor immunity	Associated with better survival Potential therapeutic target
MDSCs		/	Inhibiting the function of T cells and NK cells and weakening anti-tumor immunity	Associated with poorer survival
CAFs		Multiple subtypes Enriched in TNBC ECM remodeling and angiogenesis Immune checkpoint expression regulation	Tumor promotion Immunosuppressive microenvironment	Associated with poorer survival Drug resistance Potential therapeutic target
CAAs		Energy storage for tumor cells Expression of PDL1 Induction of cytokine secretion	Tumor growth and metastasis promotion	Unclear in TNBC

CAAs, cancer-associated adipocytes; CAFs, cancer-associated fibroblasts; DCs, dendritic cells; MDSCs, myeloid-derived suppressor cells; NK, natural killer; TAMs, tumor-associated macrophages; TILs, tumor-infiltrating lymphocytes; Tregs, regulatory T cells

Additionally, various cytokines, such as IL-6, IL-8, and TGF-β, are present in the TME of TNBC. IL-6 and IL-8 are typical inflammatory cytokines involved in tumor angiogenesis, growth, migration, and other processes, significantly promoting TNBC malignancy [103–105]. TNBC cells secrete large amounts of IL-6, which primarily acts on lung lymphocytes to activate the JAK2-STAT3 signaling pathway, inducing the upregulation of angiogenic factors and chemokines, promoting TNBC cell metastasis to lymph nodes and other structures or organs [106]. IL-8 expression is highest in the TME of TNBC compared to other breast cancer subtypes. It is secreted by monocytes and vascular endothelial cells, acting on key receptors such as CXCR1/2 to induce STAT3 phosphorylation, angiogenesis, immune chemotaxis, and tumor metastasis [104, 107, 108]. TGF-β also promotes tumor progression by inhibiting cytotoxic T cells and enhancing TAM activity in TNBC [109]. Conversely, factors like IFN-γ, IL-2, and IL-15 can have antitumor effects by stimulating M1 macrophages or NK cells [110, 111]. TNBC has a relatively high tumor mutation burden (TMB), providing an antigenic basis for immune cell recognition. PD-L1 expression is significantly elevated in the TME of TNBC, offering a promising target for TNBC immunotherapy [112]. Immunosuppressive cells and factors in the TME form a complex network influencing TNBC development, progression, and therapeutic response. Various cellular components in the TNBC microenvironment can serve as prognostic factors to predict clinical outcomes and play a positive role in TNBC treatment.

### Relationship between immunotherapy and TME in TNBC

#### Impact of TME on TNBC immunotherapy

In TNBC, there is a complex and intimate relationship between the TME and cancer immunotherapy. The TME, serving as the habitat for tumor cells, can affects TNBC immune escape, drug resistance, and tumor prognosis by modulating the functional status of immune cells and secreting various cytokines.

Immune escape is a common phenomenon in TNBC, where tumor cells evade immune system surveillance and attack through various mechanisms. If the TME is rich in inflammatory molecules, the immune system struggles to recognize and eliminate cancer cells [113]. Genetic or epigenetic changes in TNBC may lead to the upregulation of tumor-associated antigens, causing dysregulation of immune checkpoint proteins and inhibiting T cell activity via the PD-1/PD-L1 pathway, resulting in immune escape [19]. Additionally, immunosuppressive cells such as regulatory B cells, Tregs, and MDSCs in the TME inhibit effector T cells and NK cells by secreting immunosuppressive factors like IL-10 and TGF-β, promoting tumor growth and metastasis [52, 114, 115]. TAMs in the TME also contribute to immune escape by expressing immunosuppressive genes and secreting anti-inflammatory and proangiogenic factors such as VEGF [116]. Consequently, immunotherapy for TNBC has numerous potential targets in the TME, and analyzing the immune landscape of the TME can facilitate precise treatment for patients.

**Fig. 2:**
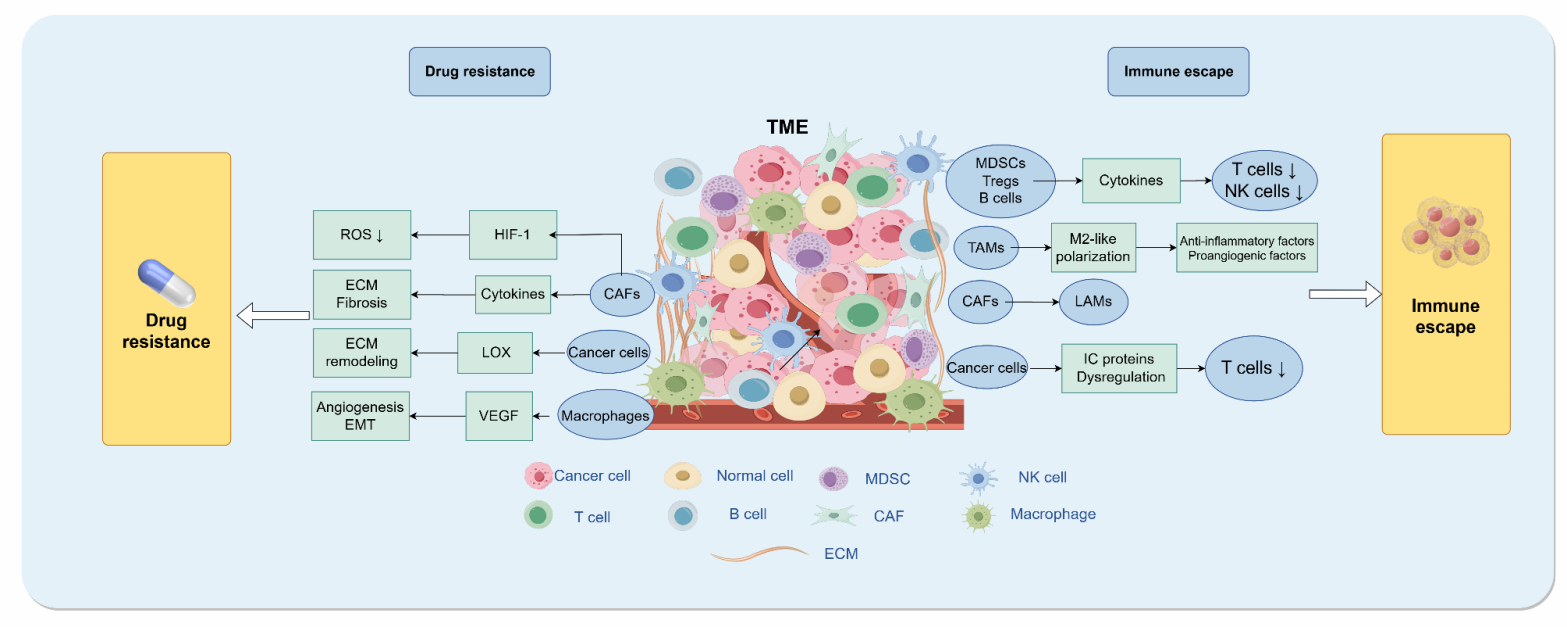
Impact of TME on TNBC immunotherapy-Drug resistance and immune escape

Drug resistance poses another significant challenge in the immunotherapy of TNBC. The resistance of TNBC tumor cells to various therapies, such as chemotherapy and immunotherapy, is intricately linked to specific components of the TME [117]. A diverse array of cells within the TME of TNBC have been implicated in the development of drug resistance. Both CAFs and M2 macrophages can not only promote angiogenesis but also increase the nutrient supply to tumor cells, fostering drug resistance through the secretion of cytokines and chemokines, such as VEGF [118–120]. Furthermore, elements within the TME can engage with TNBC cells, modifying the resistance of TNBC to drugs through metabolic changes, stress regulation, and other mechanisms. CAFs within the TME influence the metabolic processes of tumor cells by interacting with TNBC, thereby inducing resistance to anti-cancer medications [119]. The hypoxic and acidic TME resulting from the aggressive proliferation of TNBC cells also plays a role in the development of drug resistance through the activation of lysyl oxidase (LOX) and the reduction of reactive oxygen species (ROS), among other pathways [121, 122]. In addition to the interaction between components of the TME and tumor cells leading to TNBC resistance to immunotherapy, drug delivery disruptions caused by the TME are also crucial factors influencing the effectiveness of immunotherapy. In TNBC tissues, substances and cytokines secreted by CAFs contribute to severe fibrosis and extracellular matrix (ECM) deposition in the TME of TNBC, resulting in tumor vascular compression [82, 123]. This compression hinders the perfusion of tumor cells, thereby impeding drug delivery. Therefore, addressing TNBC resistance and TME remodeling is also a key focus in TNBC immunotherapy research (Fig. 2).

This figure demonstrates the complex interactions of the TME with immune escape and drug resistance in TNBC. The TME influences immune escape and treatment resistance in TNBC by modulating the function of immune cells and secreting multiple cytokines. In the immune escape mechanism, immunosuppressive cells within the TME, such as Tregs, MDSCs, and TAMs, secrete inhibitory factors, such as IL-10 and TGF-β, which weaken the anti-tumor effects of T cells and NK cells. In addition, dysregulation of the PD-1/PD-L1 pathway inhibited T cell activity. Drug resistance, on the other hand, is closely related to TME components, and CAF and M2 macrophages enhance TNBC resistance to chemotherapy and immunotherapy by secreting pro-angiogenic factors and modulating the metabolic environment. Fibrosis and extracellular matrix deposition also lead to impaired drug delivery, further weakening the therapeutic effect. CAAs, cancer-associated adipocytes; CAFs, cancer-associated fibroblasts; DCs, dendritic cells; MDSCs, myeloid-derived suppressor cells; NK, natural killer; TAMs, tumor-associated macrophages; TILs, tumor-infiltrating lymphocytes; Tregs, regulatory T cells; TNBC, triple-negative breast cancer; TME, tumor microenvironment; ECM, extracellular matrix; VEGF, vascular endothelial growth factor.

#### TME -- markers of TNBC treatment efficacy and prognosis

The TME influences the overall immune landscape of TNBC and establishes a crucial foundation for precise treatment and prognostic evaluation though modulating the functional status of immune cells and secreting various cytokines. There are some biomarkers that may help predict the treatment effect and prognosis (Table 3). The expression level of PD-L1 serves as a significant indicator of the effectiveness of immune checkpoint inhibitors. TNBC patients exhibiting high PD-L1 expression typically demonstrate improved therapeutic responses following treatment with PD-1/PD-L1 inhibitors. While, it is essential to acknowledge that PD-L1 expression levels are not absolute, as evidenced by certain lung cancer patients benefiting from immunotherapy despite testing negative for PD-L1 expression [23, 124]. Furthermore, the density and composition of TILs can be utilized as predictive factors for tumor immunotherapy outcomes. TNBC patients with high TIL density generally exhibit enhanced treatment responses and prognoses after immune checkpoint inhibitor therapy [20, 125]. Additionally, the presence of CD8 + T-lymphocyte infiltration is positively correlated with the survival duration of TNBC patients, a specific relationship not observed in other breast cancer subtypes [126]. The infiltration of Tregs is also recognized as a prognostic indicator for TNBC, with FOXP3 + Tregs indicating a poorer prognosis [127]. In TNBC, CD4 + and CD8 + T cells surround B cells to form a tertiary lymphoid structure, which can enhance anticancer immunity by delivering antigens to TILs [128]. This structure has been associated with favorable responses to immunotherapy in certain cancers. While the specific role of the tertiary lymphoid structure in TNBC remains unclear and there are no established methods for its quantification, it has been proposed that the level of B cells could serve as a prognostic indicator for a positive outcome in TNBC [129, 130]. The polarization status of TAMs also holds significant prognostic value. A higher proportion of M1-type TAMs is typically associated with improved immunotherapeutic responses, while an abundance of M2-TAMs predicts a worse prognosis [131]. In TNBC, the prevalence of M2 polarization suggests that TAM expression in the TNBC microenvironment is closely linked to poor patient prognoses [132]. Tumor-associated neutrophils (TANs) and TAMs exhibit similar predictive capabilities [133]. Besides, changes in DCs, an anti-tumor immune cell type, during treatment could help predict and monitor immunotherapy outcomes. The presence of CD103 + DCs correlates positively with clinical symptom improvement in breast cancer [134], although their role in TNBC requires further investigation.

In addition to immune cells, cytokine levels in TME and levels of substances such as antigen proteins can also be used to determine the status and prognosis of TNBC patients. As the most critical immune cells in TME, the protein levels expressed by TILs are also considered to have relevant predictive value. Specifically, increased expression of Major Histocompatibility Complex class I (MHC-I) has been linked to higher CD8 + T-cell depletion frequency. CD8 + T cells with elevated depletion markers exhibit increased expression of immune receptors like Lymphocyte-associated gene 3 (LAG3) and T cell immune receptors, along with higher levels of tumor-reactive markers such as CD39 [78]. LAG3, a transmembrane protein on TILs, acts as a negative regulator of T cell proliferation. Inhibition of LAG3, particularly in combination with PD-1 inhibition, may offer additional benefits in TNBC treatment. Thus, higher LAG3 levels may offer better treatment action, which is associated with a more favorable prognosis [28, 135]. Among different breast cancer subtypes, TNBC shows the highest expression of CTLA-4. CTLA-4 regulation by Hsa-mir-92a, forming a competitive RNA network, impacts TNBC prognosis through the T-cell activation pathway [136]. High CTLA-4 expression in breast cancer patients is linked to increased lymph node metastasis and lower survival rates, potentially serving as an indicator for immunotherapy [137, 138]. In addition to being a therapeutic target for TNBC, VEGF is also important for prognosis prediction in TNBC [82]. Elevated levels of IL-8 are associated with increased metastasis and recurrence in TNBC, while IL-18 levels are linked to poor survival outcomes in TNBC patients [104, 139]. Notably, TNBC exhibits a relatively high tumor mutational load (TMB) in the TME, providing a robust antigenic basis for immune cell recognition. TNBC patients with high TMB tend to respond better to immunotherapy, leading to improved therapeutic outcomes and prognosis [29, 112].

In addition to the biomarkers present in the TME that serve as indicators for TNBC patients, dynamic alterations within the TME are even more indicative of the impact of immunotherapy. For instance, an augmentation in the quantity and functionality of TILs within the TME after immunotherapy administration is often linked to a more favorable therapeutic outcome. With treatment, an increase in the density of TILs is often accompanied by an increase in the ratio of CD8 + to CD4 + T cells, which suggests the efficacy of the clinical treatment [72]. Variations in the levels of CAFs, MDSCs, and Tregs throughout the treatment also influence the effectiveness and prognosis of immunotherapy. Consequently, by routinely monitoring the levels of immune cells and cytokines within the TME, the efficacy of immunotherapy can be anticipated at an early stage, thereby facilitating the adjustment of therapeutic approaches [72]. Moreover, gene expression patterns and molecular markers within the TME can also serve as prognostic indicators for immunotherapy and disease progression. By scrutinizing gene expression profiles within the TME, pivotal genes and signaling pathways associated with the response to immunotherapy can be identified [140]. Utilizing single-cell RNA sequencing technology enables a comprehensive analysis of the TME’s response to immunotherapy, which can be utilized as a predictive element for assessing the patient’s prognosis [141].

**Table 3:** Potential biomarkers in the TME of TNBC

Predictive effects		Cell types		Proteins and cytokines
Better survival		Exhausted CD8 + T cells		LAG3
		CD8 + TRM-like cells		IFN-γ
		Exhausted CD4 + T cells		
		CD4 + helper cells		
		B cells		
		CD103 + DC cells		
Worse survival		Tregs		PD-L1
		TAMs		MHC
		CAFs		VEGF
		TANs		IL-8/18

CAAs, cancer-associated adipocytes; CAFs, cancer-associated fibroblasts; DCs, dendritic cells; MDSCs, myeloid-derived suppressor cells; LAG, lymphocyte-associated gene; MHC, major histocompatibility complex; NK, natural killer; TAMs, tumor-associated macrophages; TILs, tumor-infiltrating lymphocytes; Tregs, regulatory T cells; TNBC, triple-negative breast cancer; TME, tumor microenvironment; ECM, extracellular matrix; TMB, tumor mutational load; VEGF, vascular endothelial growth factor

#### Strategies for TNBC immunotherapy – targeting the TME

The TME of TNBC exhibits distinctive characteristics that play a crucial role in immune evasion and the emergence of drug resistance during immunotherapy. Consequently, numerous research studies and clinical interventions have been directed towards modulating the TME of TNBC to enhance therapeutic outcomes. These unique TME attributes not only offer a theoretical foundation but also suggest potential therapeutic strategies for implementing immunotherapy.

Immune cell-related research is a priority as a major component of TME. PD-L1 expression is notably elevated in TNBC, rendering PD-1/PD-L1 inhibitors effective in its treatment. These inhibitors have gained approval for use in various combination therapy regimens for TNBC, demonstrating efficacy. Additionally, studies on TILs have introduced novel concepts for TNBC immunotherapy. Comparing to other types of tumor, TNBC exhibits a substantial presence of TILs in the TME, which possess inherent immunogenicity, inducing anti-tumor responses. Enhanced levels of CD8 + T-cell infiltration correlate with improved prognosis [6, 142].

Another important cancer-suppressive immune cell in the TME is the NK cell. Radiotherapy has been found to induce NK cell infiltration into TNBC tumor foci [143]. PD-L1 inhibitors have also been found to enhance NK cell cytotoxicity in TNBC patients [144]. In addition to exploiting the properties of the immune cells themselves to better promote their anticancer effects or inhibit their cancer-promoting effects, the direct modification and optimization of the immune cells themselves are also gradually being explored. With the identification of several antigenic targets in TNBC, such as GD2 and CD19, chimeric antigen receptor T (CAR-T) cells created on this basis have been shown to inhibit the proliferation and metastasis of TNBC tumor cells [42, 145]. The role of CAR-NK therapies in TNBC patients is also being investigated, with chimeric antibodies being integrated into NK cells to enhance the ability of NK cells to kill cancer cells and to secrete anticancer cytokines. secretion of anticancer cytokines [146]. CIK cells, which are cultured in vitro as a group of T-diverse polyclonal immune cells expressing CD3 and CD56, exhibit NK cell-like activity and have demonstrated inhibition of cancer growth and metastasis in TNBC patients [147].

Therapeutic approaches targeting TAMs are also being explored. Lysine-specific histone demethylase (LSD) promotes TAM expression of immunosuppressive genes in the M1 direction. LSD inhibitors have been found to reduce demethylase activity in the nucleus and stimulate macrophage transformation with a decrease in MDSCs, which in turn reduces TNBC migration [148, 149].

Angiogenesis is a critical characteristic of the TME, with high expression of VEGF in TNBC. Strategies combining VEGF inhibitors with immunotherapy have shown potential in treating various solid tumors [150, 151]. VEGF inhibitors can boost immunotherapy efficacy by regulating endothelial biological status, impeding tumor angiogenesis and ameliorating the immunosuppressive TME. Also, VEGF inhibitors can enhance anti-tumor immunity by restructuring tumor vasculature to foster immune cell infiltration [152]. Bevacizumab, a VEGF inhibitor, has exhibited promising clinical outcomes in specific TNBC patients [153]. Furthermore, the functional state of immune cells and cytokine levels in the TME significantly impact immunotherapy outcomes. Some researchers propose that modulating cytokine levels in the TME, such as by blocking IL-6, TGF-β or MToR signaling pathways, can enhance immunotherapy effects [154]. Tocilizumab, an IL-6 receptor antagonist, has been shown to boost the antitumor immune response by reducing immunosuppressive cytokine secretion through IL-6 pathway inhibition [155].

With the enhanced comprehension of TME, research on tumor immunotherapy has expanded beyond specific targets. Vaccines developed against components of the tumor microenvironment of TNBC have demonstrated promising results in preclinical studies [156]. The Adagloxad simolenin vaccine, for instance, has the ability to elicit an immune response against tumor cells by targeting the Globo-H antigen, which is prevalent in the TNBC microenvironment [40]. Additionally, TNF-associated apoptosis-inducing ligand receptor (DR5) DNA vaccines, which stimulate IFN-γ secretion by T cells and induce apoptosis in tumor cells, show potential in TNBC treatment [157]. Furthermore, the utilization of DC fusion vaccines in TNBC promotes T cell expansion and the expression of immune factors, such as TL-12, which possess specific cytotoxicity against tumor cells [158]. Therapies that modify the ECM of the tumor and modulate the immunosuppressive environment to reshape the TME are also under investigation. Moreover, the efficacy of phytochemicals, nanotechnology, manipulation of gut bacteria, and immune cell manipulation in TNBC is currently being explored (Reviewed in [11]).

Currently, immunotherapy is commonly used as part of a comprehensive treatment approach for TNBC in both clinical practices and research. As discussed above, the combination of immunotherapy and chemotherapy represents a significant research direction, as we may target the characteristics of the TME to better explore the immune advantages associated with combination therapy. Chemotherapy not only kills tumor cells directly, but also enhances the antitumor effects of the immune system by modulating the TME [19]. Specifically, chemotherapy can influence the number and abundance of immune cells within the TME, promote the recruitment of TILs, and mitigate tumor immune escape by inhibiting the activity of Tregs and TAMs, as demonstrated in the TME of various tumors, including TNBC [159, 160]. Furthermore, chemotherapeutic agents can induce further conversion of the TME to a more reactive state. These agents can initiate specific T-cell immune responses by inducing immunogenic tumor cell death and promoting the release of tumor antigens into the TME [161]. Additionally, some chemotherapeutic agents have been shown to downregulate immune checkpoints, such as PD-L1 expression on tumor cells, thereby reducing immune suppression [162]. Therefore, analyzing from the perspective of TME and exploring the effectiveness of various cancer therapies in TNBC patients from the perspective of immunization will help to explore more effective combination therapies from a brand-new perspective and will help to improve the clinical efficacy of TNBC patients as much as possible.

**Fig. 3:**
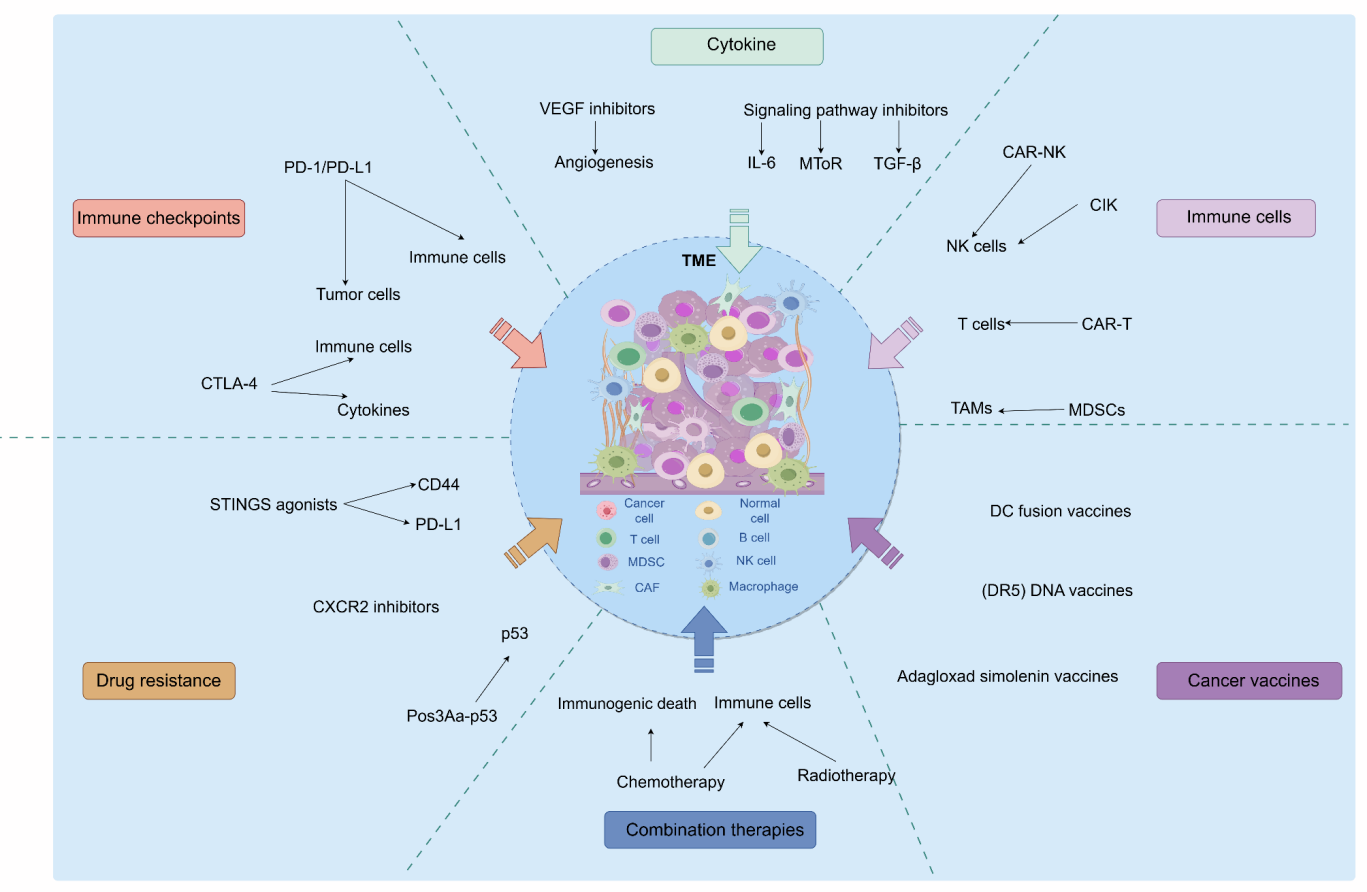
Strategies for targeting the TME in improving TNBC immunotherapy

Resistance to immunotherapy, particularly ICIs, has been extensively investigated in TNBC. Researchers are focusing on the TME characteristics to explain and overcome this challenge. TNBC patients deficient in CD8 + T cells are prone to developing resistance to PD-1/PD-L1 inhibitors, potentially due to p53 mutations or deletions [163]. Some studies have explored enhancing intracellular p53 delivery through Pos3Aa-p53 crystals, leading to increased efficacy against PD-1 inhibitors [164]. Chemokine receptor 2 (CXCR2), predominantly expressed by neutrophils and serving as a receptor for cytokines including IL-8, is notably upregulated in TNBC [165]. Combining CXCR2 inhibitors with chemotherapeutic agents or PD-L1 inhibitors has both shown promise in mitigating resistance and improving treatment outcomes [166]. Additionally, STING pathway agonists targeting CD44 and PD-L1 have been shown to increase the expression of type I interferons in the TME and facilitate the recruitment of CD8 + T cells and NK cells to tumor sites, aiding in overcoming resistance to TNBC immunotherapy [11, 167] (Fig. 3).

This figure illustrates potential therapeutic targets within the TME of TNBC and the corresponding strategies for immunotherapy. In the TME, approaches such as immune checkpoint inhibition (e.g., PD-L1 inhibitors), immune cell modification (e.g., CAR-T, CAR-NK, and CIK cells), and cytokine regulation (e.g., VEGF inhibitors) can improve the TME, enhance immune function, and inhibit tumor progression. The figure also highlights the regulatory effects of combining chemotherapy, radiotherapy, and immunotherapy on the TME. Additionally, novel immunotherapies, such as cancer vaccines, can modulate the TME to further enhance immune responses. Furthermore, immune resistance may be overcome by modulating immune cell function and cytokine levels within the TME. CAR-T, Chimeric Antigen Receptor T; CTLA-4, cytotoxic T-lymphocyte-associated protein 4; CXCR2, chemokine receptor 2; DCs, dendritic cells; ECM, extracellular matrix; MDSCs, myeloid-derived suppressor cells; NK, natural killer; PD-1, programmed cell death protein 1; PD-L1, programmed death-ligand 1; TAMs, tumor-associated macrophages; TILs, tumor-infiltrating lymphocytes; TNBC, triple-negative breast cancer; TME, tumor microenvironment; VEGF, vascular endothelial growth factor.

.

## Immunotherapy and TME in TNBC — a clinical perspective

With the increasing focus on TNBC research, the importance of identifying and utilizing the characteristic of TME in clinical settings has gained prominence. ICIs, particularly PD-1/PD-L1 inhibitors, were the initial immunotherapeutic agents integrated into the clinical management of TNBC and remain the primary immunotherapeutic agents utilized in current clinical practice. Apart from T cells, NK cells, macrophages, MDSCs, and various immune cells within the TME present promising therapeutic targets for TNBC. Additional TME components, such as angiogenesis, fibrous elements, and cytokines, also play crucial roles in tumor development and treatment, offering potential as therapeutic targets. Ongoing research endeavors aim to optimize the anti-cancer potential of these elements and restrain their tumor-promoting capabilities. Future investigations should emphasize the potential of immune cells, cytokines, and other TME components as targets for immunotherapy in TNBC, aiming to broaden treatment options and enhance therapeutic outcomes. Furthermore, novel therapies incorporating TME modulation have demonstrated significant potential in TNBC treatment. Strategies involving vaccine therapies, cellular therapies, and innovative immunomodulators not only directly target tumor cells but also enhance immunotherapeutic efficacy by modulating the TME. These therapies have shown notable efficacy in the clinical management of various solid tumors, such as melanoma and lung cancer [168], with ongoing validation of their effectiveness in TNBC treatment, potentially expanding the comprehensive clinical treatment regimen for TNBC in the future. Additionally, evolving therapeutic approaches targeting the TME, such as STING pathway agonists and CXCR2 inhibitors, as previously discussed, are emerging. Integration of nanotechnology and targeted drug delivery systems enables direct delivery of immunomodulators and anti-cancer drugs into the TME, enhancing drug distribution and bioavailability to improve therapeutic efficacy [169]. Microbiome-modulated therapies also represent a burgeoning area of research, offering novel avenues for TNBC treatment by modulating the gut microbiota and enhancing the host’s immune response [170]. These emerging therapies, in conjunction with TME-modulated approaches, present innovative strategies and promising prospects for TNBC treatment.

TNBC is one of the extensively researched cancers in the realm of immunotherapy. However, the issue of immune stratification poses a significant challenge to the application of immunotherapy in TNBC clinical practice. The intricate nature of the cancer immunotherapy process necessitates a broader range of biomarkers to accurately reflect the efficacy of immunotherapy, rather than relying solely on individual markers like PD-1/PD-L1. Conventional studies lack standardized protocols to differentiate between populations that are dominant in responding to immunotherapy and those at risk, based on marker thresholds. This limitation hinders the optimal effectiveness of immunotherapy in various clinical scenarios and does not align with the economic demands of clinical treatment [22]. Therefore, the characteristic of TME serves as a crucial focus of immunotherapy as well as holds significant potential for clinical application by serving as a key indicator for typing and prognostic prediction in TNBC. The dynamic alterations in immune cells, cytokines, and stromal components within the TME play a pivotal role in influencing the effectiveness of immunotherapy and the overall prognosis of patients [19]. For instance, biomarkers such as the expression level of PD-L1 and the quantity and type of TILs have emerged as essential indicators for predicting the therapeutic response to ICIs [124, 125]. A classification system based on TME attributes can also aid clinicians in distinguishing patients with varying immune statuses, enabling the selection of the most suitable treatment protocols. By considering factors like PD-L1 expression levels, TIL density and types, along with data from gene expression profiling and single-cell sequencing, it becomes feasible to finely categorize TNBC patients to determine the most appropriate therapeutic approach. Patients exhibiting high PD-L1 expression and high dense TILs in the TME are more likely to benefit from PD-1/PD-L1 inhibitor therapy [171, 172]. Furthermore, by integrating multiple TME markers, including levels of immunosuppressive factors (e.g., transforming growth factor-beta and interleukin-10) and VEGF, it is possible to further enhance patient stratification, thereby improving treatment precision and efficacy [173, 174]. Through the amalgamation of gene expression profiles and single-cell sequencing data, and by scrutinizing the gene expression patterns within the TME, it is plausible to identify key genes and signaling pathways associated with immunotherapy response, which can also serve as potential predictors of immunotherapy outcomes and disease prognosis in TNBC [85]. This stratified patient management can not only enhance treatment effectiveness but also minimize unnecessary treatment-related adverse effects. In cases of TNBC patients exhibiting a notable immunosuppressive state within the TME, a combination of immunomodulatory agents and conventional therapies may be necessary to enhance the efficacy of immunotherapy [175]. This personalized treatment strategy exemplifies a significant application of precision medicine in the management of TNBC.

Incorporating TME characteristics in the design of clinical trials is of significant importance as well. By integrating TME characteristics into clinical trials, the efficacy of novel drugs and therapies can be more effectively evaluated. In clinical trials assessing the effects of ICIs in TNBC treatment, treatment efficacy and patient prognosis have been judged by including PD-L1 expression levels [176]. On this premise, as previously demonstrated, numerous immune cells and immune factors within the TME hold significant prognostic value for patients. Therefore, incorporating these indicators into clinical trials could potentially offer a cost-effective and efficient means of assessing patient prognosis, leading to a more comprehensive and direct evaluation of patient status and ultimately yielding a more comprehensive outcome. Moreover, the TME profile can serve as a tool for identifying eligible patients for inclusion in clinical trials. This enhances the precision and dependability of test outcomes by pre-screening patients with inherent immune benefits prior to the commencement of the experiment [177]. By integrating TME characteristics into clinical trials, there is promising potential to expedite the advancement and implementation of new drugs and therapies, offering increased therapeutic options and optimism for patients. With further advancements in TME research, tailored treatment approaches based on TME characteristics are poised to play an increasingly pivotal role in the management of TNBC.

## Conclusion

Immunotherapy for TNBC exhibits significant potential, particularly with the use of immune checkpoint inhibitors. However, variations in effectiveness are intricately linked to the complex nature of the TME. The TME encompasses not only tumor cells but also a diverse array of immune cells (such as T cells, NK cells, and macrophages), stromal cells, blood vessels, and cytokine networks. These components collectively constitute a highly dynamic and intricate system that significantly impacts the effectiveness of immunotherapy. Specifically, enhancing the infiltration and activation of CD8 + T cells has been shown to improve the prognosis of TNBC patients. Novel immune cell therapies, such as NK cells and CAR-T cells, have also demonstrated great potential in TNBC treatment. Combinations of angiogenesis inhibitors with immunotherapy and chemotherapy have shown promising results. Modulating cytokine levels by blocking IL-6 or TGF-β signaling pathways can further enhance the effectiveness of immunotherapy. Consequently, future research should aim to comprehensively elucidate the TME, focusing on the interplay between different immune cells and molecular signals. These efforts will facilitate the discovery of novel therapeutic targets and the formulation of strategies to remodel the TME, thereby enhancing the efficacy of immunotherapy. At the same time, the TME significantly influences both the response to immunotherapy and the prognostic assessment in TNBC. Immune cells and cytokines within the TME directly reflect the immune and disease status of TNBC patients, serving as valuable biomarkers for assessing patient condition. By utilizing multi-omics analysis and a thorough examination of patient samples, it becomes possible to more accurately predict individual responses to immunotherapy. Dynamic monitoring of immune cells and their secreted cytokines in the TME can enable early prediction of immunotherapy efficacy and provide a basis for personalized treatment strategies. Thus, a deeper understanding and evaluation of the immune environment in the TME will likely drive the advancement of personalized medicine.

In conclusion, comprehending the diversity and dynamics of TNBC tumor microenvironment and deeply exploring the mechanism of TME in TNBC progression and treatment prognosis, are crucial for enhancing the efficacy of immunotherapy. Through comprehensive research and careful management of the TME, combined with immunotherapy, static and dynamic detection of biomarker components, such as cells and molecules in the TME, may enable more accurate assessments of TNBC treatment over time. This approach could improve both prognosis and quality of life for patients. Further exploration in this area is expected to transform the treatment approach for TNBC and provide valuable insights for immunotherapy in other cancer types.

## Electronic supplementary material

Below is the link to the electronic supplementary material.

Supplementary Material 1

## Data Availability

No datasets were generated or analysed during the current study.

## Abbreviations

APC: Antigen-Presenting Cells
BC: Breast Cancer
CAAs: Cancer-Associated Adipocytes
CAFs: Cancer-Associated Fibroblasts
CAR-T: Chimeric Antigen Receptor T
CTLA-4: Cytotoxic T-Lymphocyte-Associated Protein 4
CXCR2: Chemokine Receptor 2
DCs: Dendritic Cells
ECM: Extracellular Matrix
EFS: Event-Free Survival
ER: Estrogen Receptor
G-CSF: Granulocyte-Colony-Stimulating Factor
HER2: Human Epidermal Growth Factor Receptor 2
ICIs: Immune Checkpoint Inhibitors
LAG: Lymphocyte-Associated Gene
LAR: Luminal Androgen Receptor
LOX: Lysyl Oxidase
LSD: Lysine-Specific Histone Demethylase
MDSCs: Myeloid-Derived Suppressor Cells
MHC: Major Histocompatibility Complex
NK: Natural Killer
ORR: Objective Response Rate
OS: Overall Survival
pCR: Pathological Complete Response
PD-1: Programmed Cell Death Protein 1
PD-L1: Programmed Death-Ligand 1
PFS: Progression-Free Survival
PR: Progesterone Receptor
ROS: Reactive Oxygen Species
TAMs: Tumor-Associated Macrophages
TANs: Tumor-Associated Neutrophils
TIF: Tumor Interstitial Fluid
TILs: Tumor-Infiltrating Lymphocytes
TMB: Tumor Mutational Load
TME: Tumor Microenvironment
TNBC: Triple-Negative Breast Cancer
Tregs: Regulatory T Cells
VEGF: Vascular Endothelial Growth Factor
